# The role of platelets in sepsis: A review

**DOI:** 10.17305/bb.2023.10135

**Published:** 2024-08-01

**Authors:** Xinxin Xu, Yurou Wang, Yiming Tao, Wenpei Dang, Bin Yang, Yongsheng Li

**Affiliations:** 1 Department of Intensive Care Medicine, Tongji Hospital, Tongji Medical College, Huazhong University of Science and Technology, Wuhan, Hubei, China; 2 Department of Emergency, Tongji Hospital, Tongji Medical College, Huazhong University of Science and Technology, Wuhan, Hubei, China

**Keywords:** Platelets, sepsis, inflammatory, coagulation

## Abstract

Sepsis, a life-threatening condition characterized by organ dysfunction, results from a complex series of pathophysiological mechanisms, including immune dysfunction, an uncontrolled inflammatory response, and coagulation abnormalities. It is a major contributor to global mortality and severe disease development. Platelets, abundant in the circulatory system, are sensitive to changes in the body’s internal environment and are among the first cells to respond to dysregulated pro-inflammatory and pro-coagulant reactions at the onset of sepsis. In the initial stages of sepsis, the coagulation cascade, inflammatory response, and endothelial tissue damage, perpetually trigger platelet activation. These activated platelets then engage in complex inflammatory and immune reactions, potentially leading to organ dysfunction. Therefore, further research is essential to fully understand the role of platelets in sepsis pathology and to develop effective therapeutic strategies targeting the associated pathogenic pathways. This review delves into the involvement of platelets in sepsis and briefly outlines the clinical applications of associated biomarkers.

## Introduction

As defined by the Third International Consensus, sepsis is an organ dysfunction caused by an altered response to infection [1]. Its complex pathogenesis encompasses a range of issues, including immune dysfunction, an uncontrolled inflammatory response, coagulation abnormalities, and ultimately, organ failure. The management of sepsis entails measures such as controlling the source of infection, administration of antibiotics, fluid resuscitation, and provision of supportive care for associated organ dysfunction [2]. Despite the notable progress made in the treatment of sepsis in recent times, its incidence remains high and is among the main causes of critical illness and mortality globally. Moreover, there is growing awareness that even individuals who have received treatment for sepsis still suffer from long-term physical, psychological, and cognitive impairments [1].

Traditionally, platelets are recognized for their crucial role in hemostasis. However, burgeoning research indicates their significant involvement in the body’s immune response to infection [3]. Platelet count serves as a vital biomarker in sepsis, aiding in disease severity assessment in relation to platelet activation and depletion [4]. During sepsis, platelet activation is sustained due to the ongoing coagulation cascade, inflammatory response, and endothelial tissue damage [5]. The activated platelets partake in multifaceted inflammatory and immune responses, potentially leading to organ dysfunction. Despite their simple structure, platelets exhibit remarkably complex functionality. This review focuses on the role of platelets in sepsis and briefly discusses the clinical relevance of associated biomarkers.

## Platelet biology

**Figure 1.**
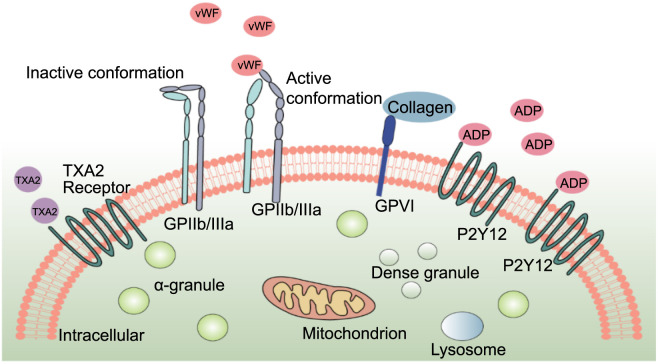
In normal circulation, platelets are in a resting state, and their most abundant membrane receptor GPIIb/IIIa (αIIbβ3) is in the so-called inactive conformation with low affinity for ligands, whereas under certain physiological or pathological conditions, the conformation of GPII b/IIIa changes after platelet activation to form an active conformation with a high affinity for ligands. Binding of TXA2, vWF, ADP, collagen, etc. to the receptor further promotes platelet activation. TXA2: Thromboxane A2; vWF: Von Willebrand factor; ADP: Adenosine diphosphate.

Platelets are biologically functional pieces of the cytoplasm shed by the cytoplasmic fragments of mature megakaryocytes in the bone marrow. Small in size and abundant in number, it is the second richest cell type in blood after red blood cells [6]. Their surfaces are studded with various adhesion and signaling molecules, such as glycoprotein IIb/IIIa (GPIIb/IIIa) integrin complexes, prostaglandin receptors (including thromboxane and prostacyclin receptors), G protein-coupled receptors (like protease-activated receptors 1 and 4 [PAR-1 and -4], and purinoceptors P2X1, P2Y1, and P2Y12), and immunoreceptors (including glycoprotein VI and C-type lectin-like receptor 2 [CLEC-2]). These molecules play pivotal roles in platelet activation and aggregation [7] (Figure 1). Platelets also contain organelles like mitochondria and various granules within their cytoplasm. The granules are of three primary types: α-granules (the most abundant, containing bioactive mediators like P-selectin, [CD62P] platelet factor 4 [PF4], coagulation factors, and high molecular mass kinins), dense granules (containing small molecules like adenosine triphosphate [ATP] and 5-hydroxytryptamine [5-HT]), and lysosomes (less abundant, holding lysosomal membrane proteins, acid hydrolases, and histone proteases). Upon activation, these granules release their contents through plasma membrane fusion, facilitating platelet functions. Additionally, platelets release extracellular vesicles into the surrounding space for intercellular communication [8].

In normal circulation conditions, platelets remain in a resting state, with a closed conformation of GPIIb/IIIa (integrin αIIbβ3), the richest membrane receptor, and low affinity for its ligands, von Willebrand factor (vWF), fibronectin, and fibrinogen [7]. In a particular physiological or pathological state, platelets are activated and undergo deformation, adhesion, aggregation, release reactions, and participate in coagulation functions. Activated platelets change their αIIbβ3 conformation and form a high-affinity open conformation, thus shifting to a high-affinity state toward both fibrinogen and additional ligands. The binding of various ligands to receptors on platelets leads to the aggregation of integrins, subsequently facilitating the outside-in signaling that drives essential platelet functions [9]. In addition, the secretion of granules from activated platelets leads to an augmentation in the levels of αIIbβ3 on the platelet membrane, platelet–platelet interactions [10]. The induction of platelet activation occurs from various sources and can be released from damaged cells, activated platelets, or inflammatory cells. Common activation inducers include thrombin, adenosine diphosphate (ADP), vWF, 5-HT, fibrinogen, fibronectin, collagen, thromboxane A2 (TXA2), and platelet-activating factor (Paf) [8].

Inhibitory mechanisms exist in the body to prevent unintended platelet activation under normal conditions and limit hemostatic reactions to vascular injury sites. Endothelial cells inhibit platelet aggregation, integrin activation, and granule secretion by secreting nitric oxide (NO) and prostacyclin (PGI2). In addition, endothelial cells express CD39, an enzyme known as phosphoryl nucleoside triphosphate hydrolase, which hydrolyzes the ADP released from activated platelets into adenosine monophosphate (AMP), thereby impeding further platelet activation [7, 11].

## Role of platelets in sepsis-induced coagulopathy

Nearly all patients with sepsis have abnormal coagulation dysfunction [12]. Sepsis-associated coagulopathy starts with the activation of sensitive markers related to coagulation factor activation, which may only be detectable initially. This is followed by a mild decline in platelet count and coagulation activation, leading to prolonged subclinical overall clotting times. Ultimately, it progresses to a more severe form known as fulminant disseminated intravascular coagulation (DIC), which is characterized by extensive microvascular thrombosis in small- and medium-sized vessels and significant bleeding from various sites.

In the context of sepsis, platelets are considered one of the first cells to react when pro-inflammatory and pro-coagulant responses are out of control because of the high number of platelets and their sensitivity to environmental changes [13]. Studies have identified that the initiation of the coagulation cascade and inflammatory response, as well as damage to endothelial tissue, constantly induces platelet activation, which increases platelet reactivity [5, 14]. Following platelet activation, several processes are associated with sepsis. The dense alpha particles fuse with the cell membrane upon activation. The ADP-rich dense granules further stimulated and amplified platelet activation through P2Y1 and P2Y12 receptors. Soluble P-selectin, a specific marker of systemic inflammation, is released from the platelet surface by binding to the P-selectin glycoprotein ligand (PSGL)-1, which regulates platelet adhesion to leukocytes and the vascular endothelium, and promotes the expression of tissue factors (TFs) on monocytes, a critical component of the exogenous coagulation pathway considered to be central to the initiation of coagulation [15–17]. Simultaneously, activated platelets may offer a phospholipid surface for the activation of coagulation factors, where the complex of activated coagulation factors aggregates on the platelet membrane, catalyzing thrombin production and making the coagulation system less susceptible to protease inhibitors [18]. Platelets also act crucially in plasma thrombin generation pathways, facilitated by histones released from dead cells, a common occurrence in sepsis [19]. Moreover, they release coagulation factors, such as fibrinogen, which convert fibrinogen into fibrin, intertwining to form clots and accelerating clotting [20]. Thrombin, the most potent platelet activator, perpetuates continuous platelet activation, leading to overactivation in sepsis [21]. Early local activation of platelets contributes to the host’s defense against pathogenic invasion at the onset of sepsis. Conversely, excessive platelet activation and abnormal coagulation enhancement can lead to in vivo coagulation dysfunction. When the coagulation system is continuously activated, massive depletion of coagulation proteins and platelets can progress to a perilous condition known as DIC. Septic DIC often gives rise to widespread microthrombosis and bleeding, which serves as a significant contributor to the occurrence of multiorgan failure [12, 16].

## Platelets and pathogens

### Platelet–bacteria interactions

Bacteria are common pathogens that interact with the platelets. As early as 1901, researchers proposed that platelets were associated with bacterial infections by demonstrating that platelets and Vibrio cholerae could form aggregates. It took decades to determine how bacteria cause platelet aggregation [22]. Many receptors interacting with bacteria on the platelet surface are expressed, for example, glycoprotein Ib (GPIb), αIIbβ3, toll-like receptor 2 (TLR2), and toll-like receptor 4 (TLR4), which can participate in platelet–bacteria interactions. In the presence of sepsis, widespread infection starts, and bacteria can activate and destroy platelets, which continue to trigger endothelial cells and exacerbate sepsis.

Studies have found that bacteria can interact with platelets through different mechanisms, including direct binding to platelet surface receptors, indirect interactions with other plasma proteins, and binding of their secretory products to platelets. This demonstrates that numerous bacteria have the ability to directly interact with platelet surface receptors, resulting in platelet activation. For instance, the fibrinogen-binding protein serine-aspartate repeat protein G (SdrG) of *Staphylococcus epidermidis* exhibits direct binding affinity toward platelet GPIIb/IIIa [23]. Similarly, the serine-rich protein A (SrpA) of *Streptococcus haematobium*, as well as the surface proteins GspB and Hsa of *Streptococcus gordonii*, demonstrate the ability to bind to GPIb in a sialic acid-dependent manner [24, 25]. In a more prevalent manner, their mechanism of action primarily involves the binding of plasma proteins, which subsequently bind to platelet receptors, as opposed to directly binding to platelets. For instance, *Staphylococcus aureus* surface protein A (SpaA) and *H. pylori* exhibit the ability to attach to vWF, subsequently facilitating an interaction with platelet GPIb [26, 27]. At the same time, the fibrin binding protein (Fnbp) on the surface of *S. aureus* [28], along with its surface clumping factors A and B (ClfA and ClfB) [29], as well as other proteins such as SdrG of *S. epidermidis* and fibrinogen-binding protein FbsA of *Streptococcus agalactiae* [23, 30], exhibit the ability to initially adhere to fibrinogen and subsequently bind to GPIIb-IIIa receptors on platelets. In numerous instances, the interaction between *S. aureus* clumping factor B and platelets is facilitated by the binding of antibodies, complement, and other factors [31]. Additionally, the secreted or shed products of bacteria exert a substantial influence on platelets. Specifically, the secretion of alpha toxins and staphylococcal superantigen-like 5 (SSL5), both originating from *S. aureus*, can directly activate platelets [32, 33]. Conversely, lysophosphatidic acid secreted by these bacteria can subsequently inhibit platelet activation via Paf receptors [34]. *Porphyromonas gingivalis* releases gingival protease, a significant virulence determinant that can induce platelet activation through the activation of PAR-1 and PAR-4, which are expressed on the surface of platelets [35].

Furthermore, bacteria can induce platelet aggregation in a manner that is different from that induced by physiological agonists (ADP and thrombin). There is an all-or-nothing response to the process, with the strongest aggregation activity occurring once a threshold concentration of bacteria is reached and no aggregation occurs below this threshold [36, 37]. Additionally, the introduction of a general soluble agonist like ADP into a suspension of platelets induces platelet aggregation within a few seconds. By contrast, the introduction of bacteria into a platelet suspension delays the aggregation response, which is concentration-dependent. The lag times observed in platelet aggregation reactions vary depending on the type of bacteria, ranging from a few minutes to 10 min [24, 27, 38–40], which is indicative of the different interactions taking place, with shorter lag times suggesting potential direct interactions and longer lag times suggesting potential indirect interactions [36].

### Platelet–virus interactions

In 1959, Danon et al. [41] observed the integration of influenza viruses into platelets using electron microscopy, initiating a new area of research focused on investigating the interplay between viruses and platelets. Thereafter, Jerushalmy et al. [42] in 1961 discovered that myxoviruses interact with human platelets in vitro. These studies provide the first indication of a direct association between viral infections and platelet counts. Studies in the following decades have found that some smaller RNA viruses such as the dengue virus (DENV), coxsackievirus (CVB), human immunodeficiency virus type 1 (HIV-1), influenza A virus (IAV), and hepatitis C virus (HCV) can bind to and internalize platelets [43–47]. In contrast, some larger DNA viruses such as herpes simplex virus type 1 (HSV-1) can bind to platelets without internalization [48]. These morphologically diverse viruses attach to and activate platelets, which exhibit direct antiviral immune activity, possibly related to the expression of many receptors interacting with the virus [49, 50]. Studies have shown that platelets express multiple pattern-recognition receptors (PRRs) that interact with viruses. Cytomegalovirus (CMV) binds to platelets through TLR2, causing platelet activation [51]. Epstein-Barr virus (EBV) interacts with platelets through complement receptor 2 (CR2) [52]. Adenovirus and hantavirus interact with platelets through GPIIb/IIIa or α2β3 integrins. Additionally, rotavirus and HCV bind to platelets via integrin α2β1 and glycoprotein GPVI, respectively. DENV and HIV, on the other hand, interact with platelets through the recognition of dendritic cell-specific ICAM3-grabbing non-integrin (DC-SIGN) receptors expressed on the platelet surface [49, 53–55]. Usually, these virus–platelet interactions mediate platelet activation, prompting their clearance by the liver and spleen. Therefore, many researchers have suggested that thrombocytopenia, frequently accompanied by viral infections, is associated with this condition [56]. In the case of SARS-CoV-2, which has become a pandemic in recent years, studies have shown that patients with SARS-CoV-2 infection have a higher degree of platelet activation, greater responsiveness, and faster aggregation [57]. Several studies have found that because both SARS-CoV and SARS-CoV-2 use angiotensin-converting enzyme 2 (ACE2) receptors to infect cells, some researchers believe that ACE2 is essential in affecting the platelet internalization of this virus, but the presence of ACE2 receptors in platelets is currently controversial [58]. In contrast, others believe that SARS-CoV-2 enters cells via transmembrane serine protease-2 (TMPRSS2), which cleaves the S protein of the virus, allowing it to fuse with the cell membrane and enter the body [59]. There is still a significant amount of uncertainty regarding the mechanisms underlying the interaction between platelets and viruses as well as the precise consequences of this interaction. Consequently, it is imperative for researchers to conduct further investigations into the involvement of platelets in viral infections, with the aim of elucidating whether their impact is advantageous or detrimental [50].

## Interaction between platelets and leukkocytes

### Platelets and neutrophils

Normally, platelets and neutrophils interact minimally in the circulation. However, during sepsis, activated platelets secrete various adhesion molecules and cytokines such as P-selectin and CD40 ligand (CD40L), which interact with PSGL-1 and CD40, respectively. This interaction mediates neutrophil recruitment, activation, and their interaction with platelets [60, 61]. The interaction between P-selectin and PSGL-1 leads to the activation of integrin macrophage antigen 1 (Mac-1), also known as CD11b/CD18, on the surface of neutrophils. Activated Mac-1 can directly interact with platelets through GPIbα, junctional adhesion molecule 3 (JAM-3), and intracellular adhesion molecule 2 (ICAM-2) [62–65]. It can also interact indirectly through fibrinogen-binding αIIbβ3 [66]. These interactions contribute to the stabilization of platelet–neutrophil interactions and play a crucial role in the subsequent activation and migration of neutrophils [67]. With the continuous activation of platelets, soluble mediators released by platelet granules, consisting of chemokines (CXCL4/PF4, CCL5/RANTES, and CXCL7) and high-kinetic histone B1 (HMGB1), further enhance neutrophil recruitment and activation. In addition, activated neutrophils can also promote platelet recruitment and activation by releasing their granular contents, such as human α-defensins (HNP 1–3), myeloperoxidase (MPO), and neutrophil peptide granular peptide cathepsin G [68–70].

Neutrophil extracellular traps (NETs) are neutrophil-derived extracellular reticulate chromatin structures composed of histones, DNA, and proteases that induce production through the interaction of activated platelets with neutrophils and serve to trap and kill invading microorganisms [71]. The interaction between P-selectin-PSGL1 and high-mobility group protein B1 (HMGB1)-receptor for advanced glycation end products (RAGE) triggers the activation of MEK-ERK signaling in neutrophils, leading to the generation of reactive oxygen species (ROS). Subsequently, ROS stimulates the activation of MPO, neutrophil elastase (NE), and protein-arginine deiminase type 4 (PAD4), ultimately resulting in the formation of NETs [72]. Recent studies have found that platelets that undergo pyroptosis during sepsis can promote the formation of NETs, which in turn induce platelet pyroptosis through positive feedback [73]. In the context of sepsis, NETs exhibit dual functions as anti-inflammatory and pro-inflammatory mediators. On the one hand, they aid in infection control, on the other, they contribute to the development of microcirculatory impairments and the infliction of tissue and organ damage [74]. Moreover, current literature suggests that the ability to monitor the formation of NETs can independently predict the occurrence and mortality of DIC in critically ill patients [75]. Thus, a better understanding of the role of NETs in disease progression in clinical applications could be effective in preventing further disease progression and, hopefully, with a deeper understanding, eventually leading to the treatment of related diseases via NETs.

### Platelets and the monocyte/macrophage system

During instances of infection or inflammation, platelets can engage with monocytes, resulting in the formation of platelet–monocyte complexes (PMCs) [76]. Certain scholars have proposed that the presence of circulating monocyte–platelet aggregates may serve as a more discerning indicator of platelet activation than the presence of P-selectin on the platelet surface [77]. Similar to neutrophils, platelets and monocytes can engage in interactions via the P-selectin-PSGL-1 and CD40L-CD40 axes, resulting in monocyte extravasation and macrophage differentiation [78]. In addition to their direct interactions, platelets possess the capability to release various chemokines that affect monocyte recruitment and differentiation. For example, platelet-released CXCL4 (PF4) can promote cytokine production and release by mediating the Janus kinase (JNK) signaling pathway and enhance monocyte phagocytosis by activating PI3K, Syk, and p38 MAPK. In addition, there is evidence that PF4 binding to IL-4 induces rapid differentiation of monocytes into antigen-presenting cells (APCs), and these differentiated APCs possess peculiar phenotypic and functional characteristics that distinguish them from conventional macrophages and dendritic cells [79, 80]. Furthermore, platelet-secreted CCL5 enhances PF4 binding to monocytes and stimulates monocyte adhesion aggregation by forming heterodimers with neutrophil-secreted human neutrophil peptide 1 (HNP1) [81, 82].

In addition to NET production, platelets can induce the generation of extracellular traps (ETs) by monocytes and macrophages, also known as METs [83]. The morphology of the ETs formed by monocytes and NETs exhibits significant similarities, as they are associated with MPO, elastase, and other factors. However, the release of monocyte ETs is not dependent on MPO activity, but rather on an oxidative burst [84]. Unlike the extensive research conducted on NETs, investigations of monocyte ETs in the context of inflammation and infection are relatively limited, and further exploration is necessary to understand their mechanisms of action in diseases. In 2010, researchers first reported that macrophages could produce ETs. Subsequently, other studies have shown that macrophages can produce METs of different origins and that the polarization of macrophages may influence MET formation, with M1 macrophages being more likely to promote MET formation [85–87]. METs, which bear a structural resemblance to NETs, have been found to be associated with the NADPH/ROS system in terms of their formation mechanism, although they can also occur independently of NADPH/ROS [86, 88–90]. Recent investigations have demonstrated that METs not only function as pathogen scavengers but also potentially contribute to tissue damage exacerbation and inflammation promotion. Consequently, further comprehensive research is imperative to enhance our understanding of the METs.

### Platelets and T lymphocytes

The types of T lymphocytes are numerous and broadly divided into two subtypes: CD8+ and CD4+T cells. Infected, cancerous, and senescent cells are recognized and killed by CD8+T cells, also known as cytotoxic T lymphocytes (CTLs). CD4+T cells, also called helper T cells, play a central role in the secretion of cytokines and activation of other immune cells. Infections can occur in all body parts, but T cell antigen presentation and activation can only occur in lymphoid tissues [6]. Circulating T lymphocytes can enter lymph nodes through small high endothelial veins (HEV) within lymph nodes, and activated platelets have been shown to combine with circulating lymphocytes and mediate the rolling of T lymphocytes in HEV via P-selectin [91].

Furthermore, a study using a hepatitis model found that platelet activation significantly contributed to CTL aggregation at sites of inflammation [92]. Among others, an experiment by co-culturing human CD4+ T cells and autologous platelets in vitro showed that platelets could influence Th1, Th17, and regulatory T (Treg cells) differentiation and associated cytokine production and differentially regulate different CD4+ T cell subpopulations, providing evidence that platelets can mediate through direct cell–cell contact and the release of multiple chemokines, CD4+ T cell differentiation, and cytokine production [93, 94]. In addition, platelets regulate T cell activity and function. During inflammatory episodes, platelets are recruited to the lungs along with neutrophils, and again to Treg cells during the receding phase of inflammation. The prerequisite for lung recruitment is the formation of platelet–Treg cell aggregates, which are associated with platelet expression of factors such as PF4, P-selectin, and CD40. The presence of platelet–Treg cell aggregates is necessary for the polarization of macrophages toward an anti-inflammatory phenotype. Moreover, the interplay between platelets and Treg cells is of utmost importance in regulating the release of anti-inflammatory cytokines, namely interleukin-10 (IL-10) and transforming growth factor beta (TGFβ), by T cells. The resolution of inflammation relies heavily on effective communication between these cellular entities [95]. In addition, bearing in mind that even the same platelet mediator may have different effects on different T cell subsets, which may be related to specific organismal microenvironments. Platelets exert an influence on T cells, while reciprocally, T cells can also affect platelet function through the release of cytokines, including interferon-gamma (IFN-γ) and interleukin-2 (IL-2). IFN-γ plays a role in promoting platelet activation, augmenting leukocyte adhesion, and inducing cytotoxicity [96, 97]. IL-2 exerts an indirect inhibitory effect on platelet aggregation by acting on monocytes, subsequently triggering platelet secretion following the inhibition of aggregation [98]. The effects of platelets on T lymphocytes include both direct and indirect effects. Nevertheless, the precise mechanisms underlying the platelet-mediated transportation, differentiation, and activation of T cells remain elusive and require further comprehensive investigation.

## Platelet-derived extracellular vesicles

Activated platelets release platelet extracellular vesicles (PEVs), which facilitate intercellular communication via substance exchange. Two main types of PEVs have been identified: particles with 100 nm to 1 mm diameter and exosomes with 40–100 nm diameter. Those formed by budding from the cell surface plasma membrane are particulates; therefore, they have the same antigens as platelets and usually contain proteins, multiple RNAs, and organelles. Another type of endosomal origin is exosomes, and endosomal PEVs contain mRNA, miRNAs, and proteins [8, 99]. Platelets directly or indirectly regulate coagulation and inflammatory responses during sepsis via PEVs. Several studies have found that the procoagulant capacity of PEVs is 50–100 times greater than that of activated platelets [100], which is associated with an increased area of exposed phosphatidylserine on the surface of PEVs [101]. During infection, platelets induce immune responses in the host that may be mediated by PEVs. PEV can promote inflammatory responses by directly recruiting monocytes, T cells, and other leukocytes, and releasing chemokines. Additionally, PEV facilitates the interaction between monocytes and endothelial cells by binding to P-selectin and PSGL1 [102]. Furthermore, certain pathogens, such as *S. aureus*, whose surface-specific antigen SSL5 can stimulate the release of PEV by binding to platelet membrane receptors, specifically the glycoproteins GPIba, GPIIb/IIIa, or GPVI. The released PEV enhances their interaction with monocytes through the CD40L-CD40 response and P-selectin-PSGL1 axis, thereby promoting the release of inflammatory mediators [103]. In the context of infection, other pathogens such as *Neisseria meningitidis* can induce increased TF expression through PEVs, thereby activating the coagulation cascade associated with sepsis [104]. Presently, platelet-derived exosomes, in addition to platelets, have been shown to promote excessive NET formation in sepsis and organ injury [105]. In addition, several studies have observed alterations in the levels of PEVs in sepsis, leading to the speculation that these vesicles may serve as novel clinical biomarkers for predicting disease progression and severity. Notably, these vesicular structures are small in size and exhibit a remarkable ability to target injury sites across various physiological barriers within the body. Consequently, the rational utilization of these structures in the development of new drug delivery systems holds great promise for enhancing the efficacy of sepsis treatment. However, the precise mechanisms by which these vesicles contribute to disease development remain unclear. Hence, it is imperative to proactively investigate and elucidate its mechanism of action to optimize its application in the diagnosis and treatment of clinical ailments.

## Platelets and endothelial cells

In the absence of any abnormal conditions affecting the organism, the endothelium remains undamaged and platelets typically do not engage with it. However, in sepsis, a condition characterized by infection and organ dysfunction, endothelial cells are in the face of substantial damage. Consequently, platelets adhere to the compromised endothelium, assuming a multifaceted role in conjunction with the exposed subendothelial structures, ultimately resulting in the formation of platelet aggregates [106, 107]. Generally, the endothelium of blood vessels covers a structure called the glycocalyx, which is composed of proteoglycans, glycosaminoglycans (GAGs), and plasma proteins (like albumin and antithrombin). It also prevents unwanted intercellular adhesion and exerts anti-inflammatory, vascular permeability-regulating, and prothrombotic effects. During sepsis, ROS, proteases, and other substances destroy the glycocalyx, one of the earliest and most important sites of injury when sepsis occurs. The disruption and detachment of this structure lead to the exposure of adhesion molecules on the endothelium, and the subsequent exposure of adhesion molecules promotes leukocyte and platelet recruitment, which in turn leads to thrombus formation [108, 109]. Platelet–endothelial interactions are facilitated via cell surface receptors, namely selectins and integrins, as well as adhesion proteins such as vWF, and occur through diverse mechanisms. As the initiating event of adhesion, platelets roll along the endothelium, and the primary mechanism is through the interaction of the platelet receptor GPIb-IX-V with vWF released in significant quantities by the endothelium, during which the P-selectin/PSGL-1 axis formed between the platelets and the endothelium plays a supporting role. Afterward, αIIbβ3 on platelets further stabilizes and enhances platelet–endothelial interactions by interacting with αvβ3 on endothelium and intercellular adhesion molecule 1 (ICAM-1) [110]. Additionally, platelets possess the capability to influence the functionality of endothelial cells through the release of various cytokines and chemokines. Research has demonstrated that activated platelets release IL-1β, which serves to enhance the expression of endothelial adhesion molecules and stimulate pro-inflammatory responses, as well as the secretion of chemokines. Moreover, activated platelets facilitate the deposition of chemokines such as CCL5 and PF4 onto the surface of endothelial cells, thereby promoting the recruitment of leukocytes. Furthermore, platelet-derived CCL5 and PF4 heterodimers have been found to augment the ability of lung tissue to attract neutrophils [111, 112]. Other studies found that liver regeneration is associated with platelet–endothelial interactions [113]. Extensive microthrombosis, inadequate tissue perfusion, associated coagulation dysfunction, and multiorgan failure during sepsis are inextricably linked to endothelial disruption and platelet–endothelial cell interactions. Both these factors are also implicated in the uncontrolled inflammatory response observed during sepsis. Therefore, it is imperative to actively investigate the mechanisms underlying these phenomena.

## Platelets and endocrine system

Regular activity and energy metabolism are closely related to the endocrine system. The normal stress response of the body regulates changes in function in response to various stressors. However, aberrations in this response occur during sepsis, leading to an imbalance. However, the significance of the endocrine system as a pivotal determinant of organ dysfunction and immunosuppression in sepsis has been largely disregarded [114]. During the early stages of sepsis, the levels of cortisol, catecholamines, and other substances are elevated in response to severe stress. Several years ago, researchers proposed that increased adrenaline secretion in response to severe stress could result in increased platelet activation [115, 116]. In addition, a study conducted on trauma patients showed that short-term co-incubation of platelets with epinephrine enhanced platelet aggregation, adhesion, and activation of GpIIb/IIIa. In contrast, long-term co-incubation reduces platelet adhesion, aggregation, and expression of GpIIb/IIIa [117]. More recently, researchers have discovered a negative correlation between plasma cortisol levels and platelet reactivity in elderly individuals [118]. Furthermore, research has also revealed that the platelets express the circadian protein Rev-erbα and enhance platelet activation and thrombosis [119]. At present, investigations on platelet interactions with endocrine-related substances in sepsis remain limited, and further studies are needed to elucidate how these endocrine hormones interact and mediate the series of pathological changes that occur in sepsis.

**Figure 2.**
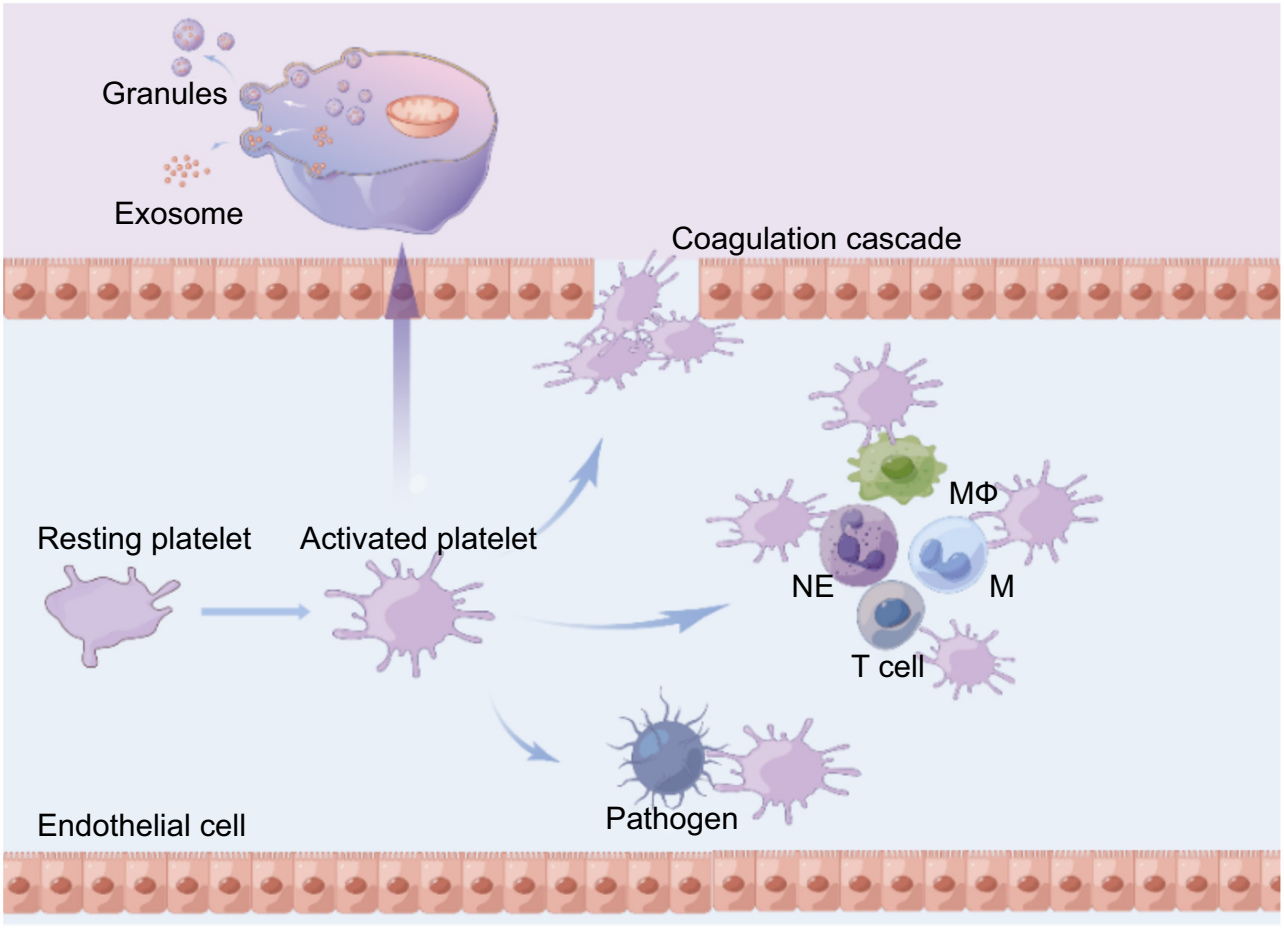
**Endothelial damage during sepsis and various other factors such as pathogens, activate platelets.** Activated platelets can interact with pathogens, neutrophils, monocytes, and endothelial cells through self-expressed receptors or by releasing various granules and exosomes, among other things, contributing to the excessive inflammatory response and various coagulation abnormalities that occur during sepsis. NE: Neutrophil; M: Monocyte; MΦ: Macrophage.

**Table 1:** Some platelet-related metrics in clinical applications

**Clinical indicator**	**Function**	**References**
Platelet count	Decreased counts suggest increased risk of death	[122, 123]
Altered platelet morphology (MPV, PDW)	Elevated MPV and PDW associated with higher mortality rates	[124, 125]
Platelet mitochondrial function	Mitochondrial dysfunction correlates with poor prognosis in sepsis	[126]
Platelet aggregation rate	Predictive value for early identification of sepsis patients at high risk of death	[126]

MPV: Mean platelet volume; PDW: Platelet volume distribution width.

## Clinical application of platelets in sepsis

Platelet abnormalities frequently occur in patients with sepsis and correlate with poorer prognosis. Platelet count, morphology, and function are significant predictors of risk stratification in patients with sepsis [120] (Table 1). As part of the sequential organ failure assessment (SOFA) score for sepsis, the platelet count is highly correlated with sepsis severity and prognosis [121]. Decreased platelet counts have been reported in ICU units and may indicate an increased risk of death in patients [122]. Moreover, in a study of patients admitted to the ICU with severe pneumonia, the lower the platelet count on admission, the higher the probability of septic shock and death after entry [123]. In addition to platelet count, morphological alterations in platelets are widespread and prognostically relevant in sepsis. Changes in mean platelet volume (MPV) and platelet volume distribution width (PDW) commonly reflect morphological alterations in platelets. MPV and PDW levels are elevated in sepsis, and increased MPV after hospital admission is independently associated with higher mortality in critically ill patients [124, 125].

Platelet mitochondrial dysfunction proves the poor prognosis of sepsis, and recent research has indicated that impaired platelet mitochondrial activity affects platelet aggregation and is correlated with sepsis severity. Studies have also indicated that the platelet aggregation rate has the potential to be an early predictive biomarker of sepsis mortality and has predictive value for the early identification of patients with sepsis who are at high risk of death [126]. As the field of research advances, there is a growing recognition of the expanding functions of platelets. Consequently, it is imperative to develop more sophisticated testing methodologies to evaluate the diverse effects of platelets comprehensively. This will enable a more comprehensive understanding of the role of platelets in sepsis, as well as the reciprocal effects of sepsis on platelets.

Given that platelet activation worsens coagulation and inflammatory responses in sepsis, resulting in organ dysfunction, there is a need to explore the potential benefits of antiplatelet agents in improving the prognosis of sepsis. Despite the ongoing debate surrounding the use of antiplatelet therapy, numerous studies have provided evidence of its advantages in patients [127]. Aspirin and clopidogrel are commonly used antiplatelet agents. A retrospective study conducted by Eisen et al. [128] revealed a significant association between the use of acetylsalicylic acid (ASA) and the survival of patients with sepsis in the ICU. Moreover, a meta-analysis of 6823 patients hospitalized for sepsis between 2011–2016 between aspirin use and mortality noted a reduced risk of death in patients who received aspirin alone before the onset of sepsis [129]. Ouyang et al. [130] also concluded in a meta-analysis that using antiplatelet agents reduced mortality in patients with sepsis. Several cohort studies have recently shown that prehospital aspirin therapy reduces sepsis-related mortality [131, 132]. Of course, in addition, there are many other drug targets that have been proposed for antiplatelet therapy, such as FcRIIA signaling inhibitors, complement inhibitors, calpain inhibitors, leukotriene receptor antagonists (montelukast), vasodilators (NO), Bruton’s tyrosine kinase (BTK) inhibitors (Ibrutinib, Dasatinib, etc.), and GPIIb/IIIa receptor blockers (Eptifibatide, Tirofiban) [133–140].

Nevertheless, more randomized controlled trials are lacking to validate whether antiplatelet agents improve the prognosis of sepsis to assess the actual efficacy of antiplatelet therapy in clinical applications.

## Conclusion

In conclusion, platelets play a pivotal role in the pathogenesis of sepsis, and the mechanisms underlying platelet activation in sepsis are intricate, encompassing the inflammatory, thrombosis, and immune responses. A comprehensive investigation of the interactions between activated platelets, their secretory products, immune cells, pathogens, and endothelial cells is essential for gaining valuable insights into the diagnosis and management of sepsis (Figure 2). As fundamental research on platelet function continues to advance, there is a need to enhance and diversify the application of testing techniques in clinical settings. This includes focusing on the investigation of combined indicators and the monitoring of platelet secretory function, among other aspects, to enhance the prediction of prognosis in patients with sepsis and deepen our understanding of its pathogenesis. The efficacy of anti-platelet therapy for ameliorating sepsis remains controversial, although animal experiments and clinical studies have demonstrated its potential. However, further research is necessary to fully elucidate its effectiveness.
